# Two window-minimally invasive lumbar spine surgery (new approach) has a better post operative outcome and less soft tissue damage

**DOI:** 10.1016/j.amsu.2020.04.037

**Published:** 2020-05-12

**Authors:** Gemah Moammer, Yasir Rehman, Sameh Abolfotouh

**Affiliations:** a McMaster University, Grand River Hospital, Kitchener, ON, Canada; b Health Research Methodology, McMaster University, Hamilton, ON, Canada; c OrthoCure Medical Center, Dubai, United Arab Emirates

**Keywords:** TLIF, Minimally invasive, Two window, Surgical outcome

## Abstract

**Introduction:**

The purpose of this new approach is to develop a method that is less invasive as well as less traumatic and can provide a better exposure/view of the surgical field. Postoperatively, the patient has less pain, short hospital stay and less use of the postoperative pain control medications. As compared to other minimally invasive spine surgeries this approach results in less soft tissue damage, minimal muscle destruction, less retraction and better surgical outcome.

**Methods:**

In this article authors focus on the new approach that has cost effective benefits as well as short recovery time postoperatively.

**Results:**

Approach is applicable for severe spinal stenosis as compared to other Minimally Invasive Spine Surgery (MISS) techniques that are only applicable for the mild to moderate stenosis or degenerative processes. This plane is avascular plane so no or less bleeding is anticipated from this procedure.

**Conclusion:**

The technique facilitates bilateral canal enlargement through unilateral approach and provides accessibility to the contralateral foramen for decompression with perfect exposure and allows instrumentation through the lateral window with no muscle destruction.

## Highlights

• Minimally Invasive Spine Surgery, Fusion, Two windows approach.

## 1 Introduction

Spine surgeries are continuously going through the process of modifications. Open Transforaminal Lumbar Interbody Fusion (TLIF) is associated with long-term convalescence, prolonged general anesthesia, and wide dissection of tissue that can cause bleeding, scarring and eventual destabilization of spinal segments [1]. The frequent use of retractors to expose the anatomic landmark for pedicle screw insertion that can cause muscle fibers damage, large doses of combined pain killers including narcotics which all affect the post-operative mobilization of the patient and increases the hospital stay [[2], [3], [4], [5]]. Minimally invasive spine surgery (MISS) was developed and has gone through continuous modifications. Its indications are expanding to decreases the approach related complications, with benefits including smaller incisions, less tissue trauma and improved outcomes [2,[6], [7], [8], [9], [10]]. It is found that although the approach is different than the open approach, the outcomes are equally effective with rapid recovery rate, decrease pain and time required to return to work [[11], [12], [13]].

There are different techniques of MISS i.e. conventional MISS and endoscopic MISS (pioneered by Lyman Smith, Hijikata, Parviz Kambin, Adam Schreiber, and HJ.Leu) [1]. The conditions requiring the decompressive surgery are degenerated spine disease, disc herniation, spinal stenosis, fusion for degenerative spinal disorders, other conditions neural compressions, vertebral body fractures and spinal tumors [1,6].

The work has been reported in line with the STROCSS criteria [14]. It has also been submitted for registration to the Research Registry with identifying number: researchregistry5510.

This is a description of a novel surgical technique description and is exempted from ethical approval in our institution.

## 2 Surgical technique

**Fig. 1:**
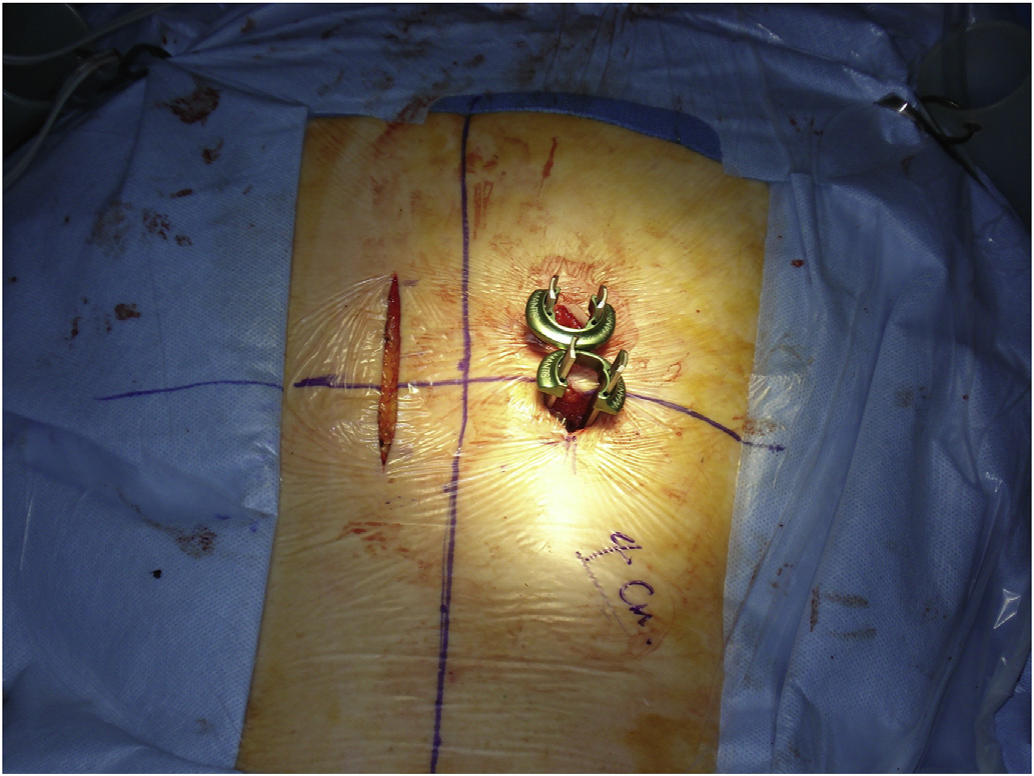
A skin incision 1 cm lateral to the center of the pedicle.

**Fig. 2:**
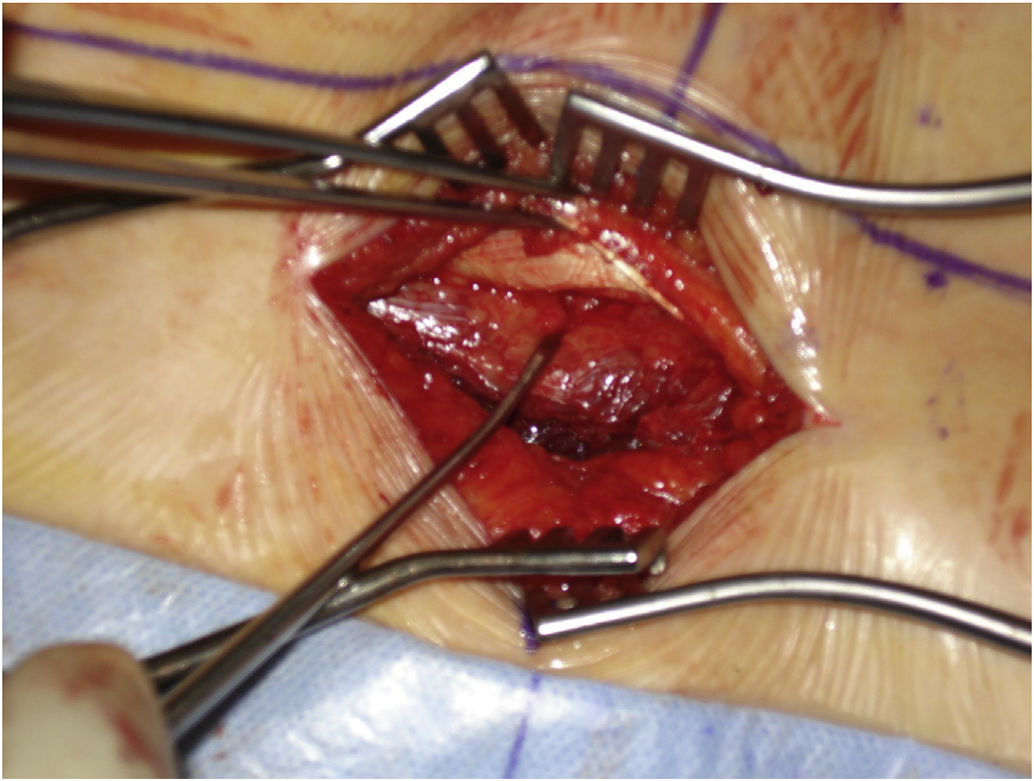
Fascial incision at the lateral border of the multifidus muscle and using a blunt dissection down to the intramuscular plane between the longissmus muscle and the multifidus muscle.

**Fig. 3:**
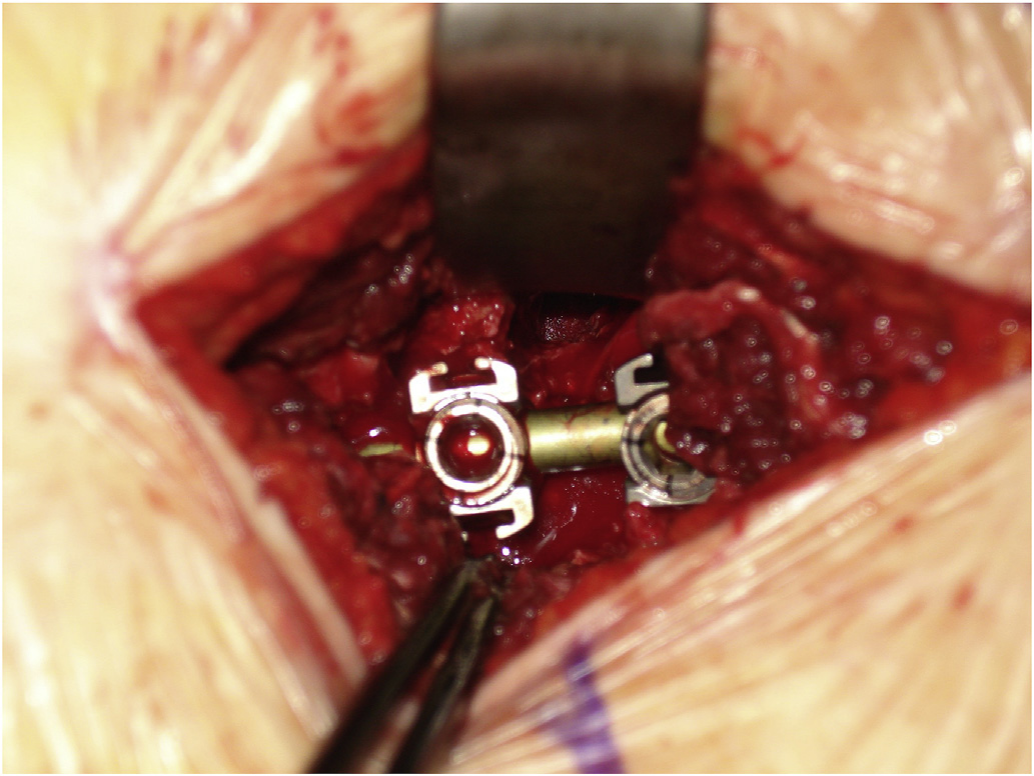
Feel the lateral aspect of the facet joint above and below and this is the lateral window of the approach.

**Fig. 4:**
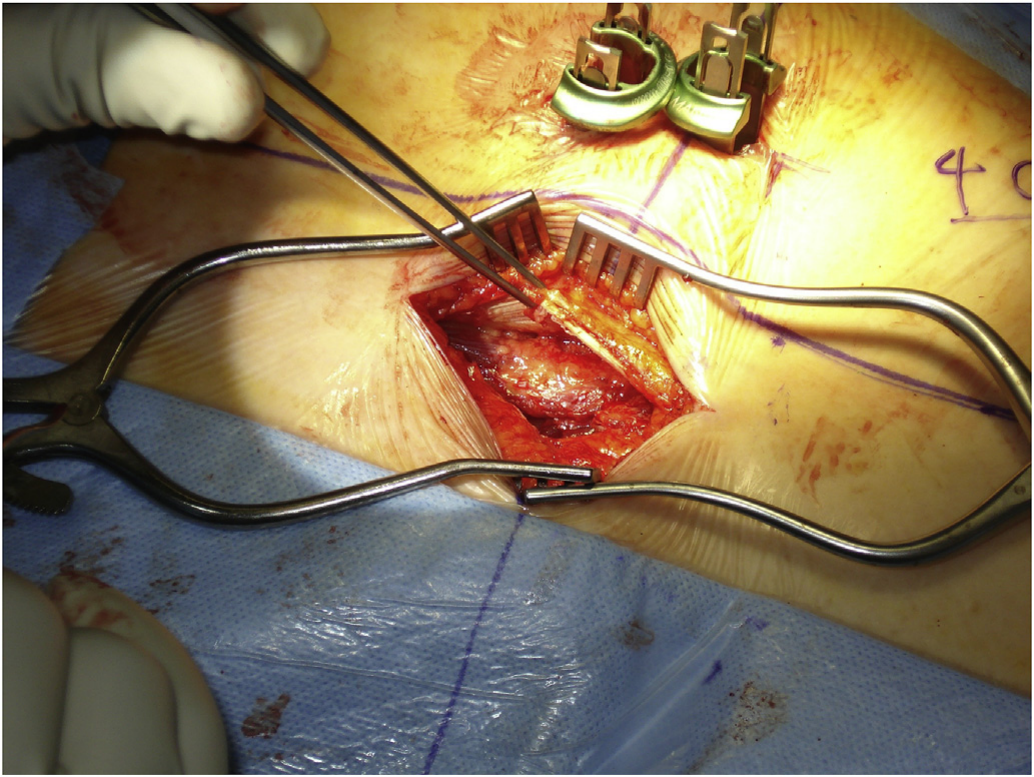
Creating the medial window by doing undermining of the medial lumboscaral fascial flap bluntly dissecting the multifidus muscle of the underneath surface of the fascial flap.

**Fig. 5:**
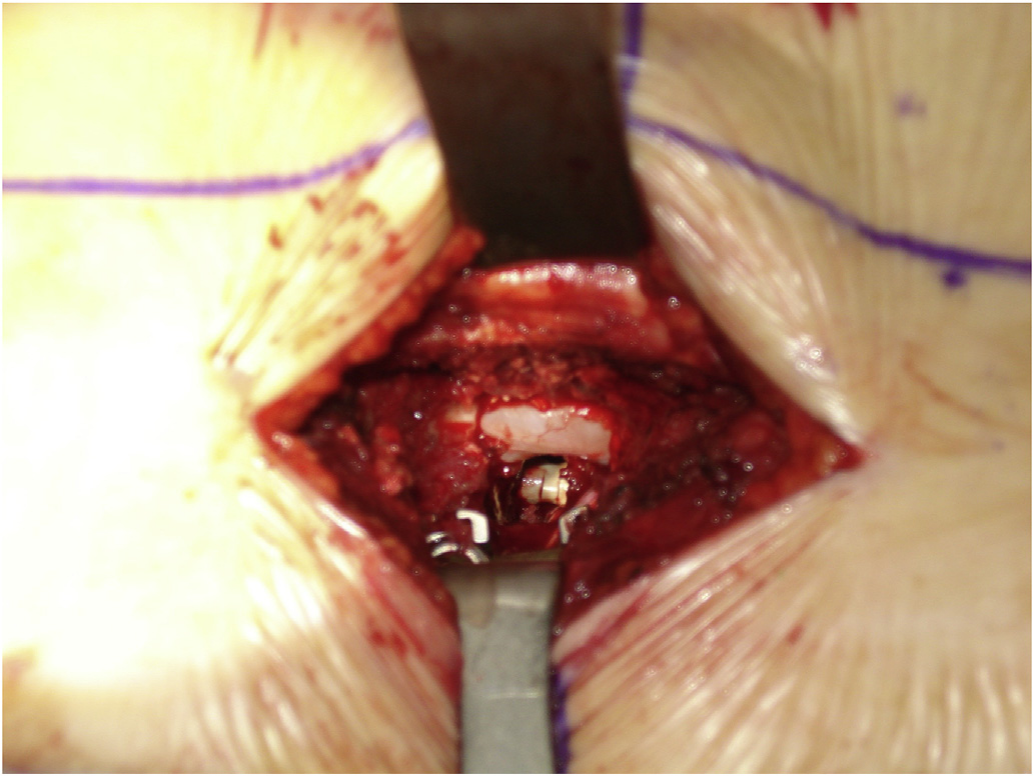
Subperiosteal elevation of the multifidus muscle of the spinous process following the approach all the way to the lamina and laterally to the facet joint.

**Fig. 6:**
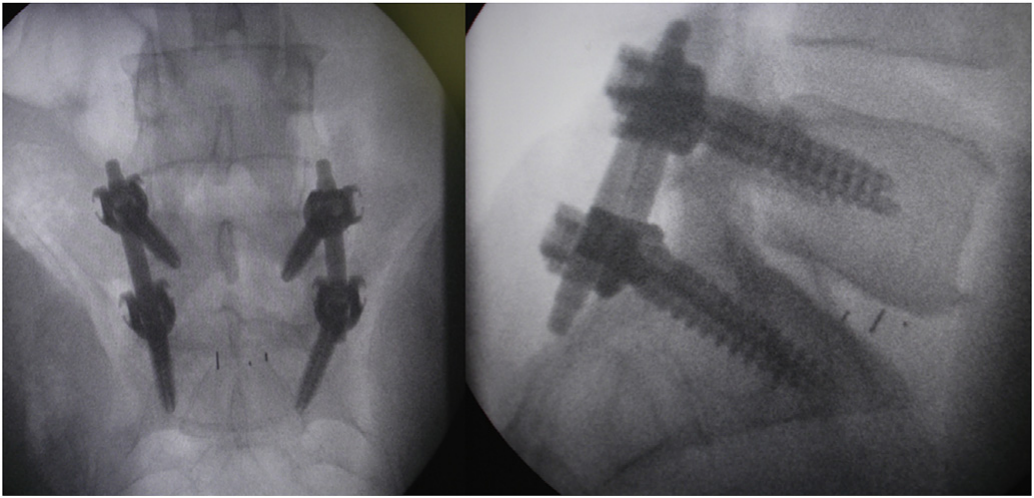
Intraoperative, x-ray confirming hardware placement.

**Fig. 7:**
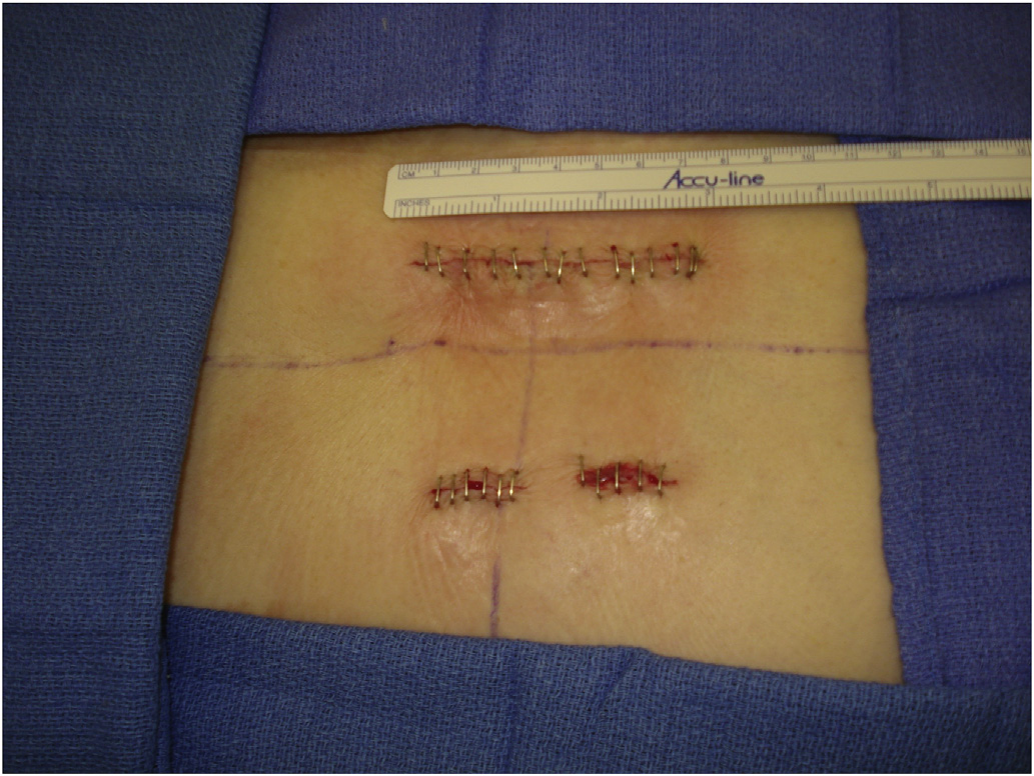
Wound closure in layers.

Mark the midline along the spinous process around the surgical/involved area and also mark the center of the contralateral pedicle. Make 1 cm incision just 1 cm lateral to the center of the pedicle, subcutaneous fascia and deep fascia, incise in same line of skin incision as well as same length (Fig. 1). Jamshedi (Biopsy needle) under imaging intensifier is inserted to get starting point at the superior lateral corner of the pedicle and proceed toward the center of the pedicle. At this point one can get a lateral view as well as view for aiming toward the right direction right in the middle of the pedicle on the lateral view. After getting jamshedi all the way down to the appropriate depth of the screw then just switch the Jamshedi over a K-wire and use the tap of a 1 mm small diameter than your planned screw insertion. Get the depth of screw and insert the appropriate screw. Insert the 2nd screw on the same side using the same technique through a different 1 cm incision then switch to the other side for the mini opening. After measuring the distance (4–4.5 cm; average is 2–2.5 cm) from the midline to the incision is made for the contralateral screw, the mini incision will be made laterally half of this distance which is around and the length of the incision will match the same length from the bottom end of the upper screw insertion incision down to the upper end of the lower screw insertion incision. Make the skin incision as template. Subcutaneous fascia and deep fascia should be incised in the same line of skin incision. At this level here the fascial incision will be at the lateral border of the multifidus muscle and using a blunt dissection following the lateral border of the muscle will lead you down to the intramuscular plane between the longissmus muscle and the multifidus muscle (Fig. 2). Carry out the planned dissection all the way anteriorly until the tips of the transfers’ processes are felt. A level above and the level below, you should be able to feel the lateral aspect of the facet joint above and below and this is the lateral window of the approach (Fig. 3). The medial window is created by doing undermining of the medial lumboscaral fascial flap bluntly dissecting the multifidus muscle of the underneath surface of the fascial flap (Fig. 4) moving towards the midline along the whole length of the incision until the tips of the spinous process of the level above and the level below are felt. Then using the monopolar/cautery do subperiosteal elevation of the multifidus muscle of the spinous process following the approach all the way to the lamina and laterally to the facet joint as the classic approach for the unilateral spinal decompression approach. By which the medial window is created (Fig. 5). Using the appropriate retractors is very important to help reduce the muscle retractions. After creating the two windows, move to the lateral windows, use the muscle retractor, expose the screw insertion under direct vision for the screw above and the screw below and do screw insertion to the interested level and then move to the medial window to start the decompression using high speed burr for the laminectomy and the undercutting of the spinous process to be able to access to the contralateral side. After finishing the decompression and exposing the Dura as well as the exiting nerve root, gently retract the nerve root and the Dura towards to the midline to expose the disc. Do a discectomy and then insert the interbody space distracter through the lateral window to the screw heads and create a minimal distraction to allow you to prepare the endplates of this interval. Partial facetectomy is required most of the time and some time total facetectomy to be able to do a full decompression and easy TLIF insertion. After the insertion of the TLIF, release the distraction, apply the rods to the bilateral sides and do final tightening of the rods with compression of the TLIF level, copious irrigation to the surgical feed and then through the lateral window, do decortications to the transverse process above and below and apply the bone graft. At this stage, intraoperative, x-ray is required to confirm hardware placement (Fig. 6), and then finish your closure in the usual technique (Fig. 7).

## 3 Discussion

The classic technique for MIS lumbar spine decompression and TLIF (contralateral side percutaneous screw insertion and ipsilateral mini open technique for a decompression and percutaneous screw insertion) are techniques for a mild to moderate spinal stenosis, unilateral/lateral recess or unilateral foraminal stenosis. More severe spinal stenosis with bilateral lateral recess or foraminal stenosis will make the tube decompression technique more challenging and will increase the risk of complications.

In conventional MIS technique if incision is more lateral it gives easy access for pedicle screws insertion but will make central canal decompression and far lateral & contralateral foraminotomy more challenging, requiring lots of muscle elevation and retraction to the midline. If the incision is more towards the midline, it facilitates the decompression but screw insertion will be challenging as lateral traction of the muscles is required, as well cage insertion will be challenging due to close proximity to midline rather than laterally which is the classic insertion point for the cage.

The new minimally invasive surgery (two window technique for lumbar spine TLIF) addresses all of these challenges and applicable for severe spinal stenosis and multiple levels with easy midline approach to decompress the canal and access to the far lateral and contralateral aspect for screw and cage insertion.

The medial window is exactly the same as used in classic unilateral approach decompression, discectomy and laminoplasty by doing the McCullough procedure, the lateral window (the lateral side of the multifidus muscle) between the multifidus muscle and the longissmus muscle gives access all the way down to the lateral aspect of the facet joint and the transverse process will allow to insert the pedicle screws very easily and to get good access to TLIF. The surgical plane is avascular plane that minimizes the blood loss, facilitates bilateral canal enlargement and instrumentation through unilateral approach and accessibility to contralateral foramen for decompression.

Percutaneous pedicle screw fixation and decompression through tubular retractors also has a limitation for the multilevel lesion or pathology, Also Disc collapse, significant spondylolisthesis and high sacral slope make more challenges [14], as well as the use of more complicated instruments i.e. radiolucent and rotating table, tubular retractor system, c-arm, microscope, percutaneous pedicle screw system, use bone collector on suction [15]. During the surgery vision may be obstructed due to use of tools in a narrow space [15]. There is also cost effective difference too i.e. use of the high performance microscopes and use of the fiberoptic light source. Another limitation of the surgery are that due to the narrow space recurrence of the symptoms can occur very easily even with a minor small hematoma [15].

## 4 Conclusion

The new minimally invasive two window approach is an effective way for transforaminal interbody fusion surgery with the same surgical outcome and fewer complications than the traditional tubular minimally invasive approach.

## Consent

Written informed consent was obtained from the patient for publication of this case report and accompanying images. A copy of the written consent is available for review by the Editor-in-Chief of this journal on request”.

## Ethical approval

Study is exempt from ethical approval in my institution.

## Funding

No Source of funding.

## Author contribution

Sameh Abolfotouh: Manuscript revision/editing and Author of correspondence.

Gemah Moammer: Surgeon and manuscript writing.

Yasir Rehman: Manuscript writing and literature review.

## Research registration number

ResearchRegistry5510

https://www.researchregistry.com/browse-the-registry#home/?view_2_search=5510&view_2_page=1.

## Guarantor

Gemeh Moammer, MD, FRCS (C).

## Provenance and peer review

Not commissioned, externally peer reviewed.

## Declaration of competing interest

No Conflict of Interest.
